# Supplementary material for: Efficient and robust coding in heterogeneous recurrent networks
Source: PLoS Comput Biol. 2021 Apr 30;17(4):e1008673. doi: 10.1371/journal.pcbi.1008673 (PMC8115785; doi:10.1371/journal.pcbi.1008673)
Supplement: S1 Text — Section A: A word on notation. Section B: Technical notes. Section C: Efficiency measures, including Supplemental Figures A and B. Fig A: Comparison of efficiency measures Results of two simulations using the same stimulus, but different initial network states, in a homogeneous ‘type 1’ network (first row), a network with ‘type 1’ and ‘type 2’ neurons (second row) and a heterogeneous network (third row). Three alternative efficiency measures are compared: 1) the network efficiency multiplied by the stimulus amplitude (Ea, see supp. eq. (10), first column), 2) the network efficiency multiplied by the stimulus power (Ea2, see supp/ eq. (11), second column) and 3) the network cost (C, see supp. eq. (12), third column). The bottom row shows how the network efficiency or cost depends on the spike reliability Γ¯. Parameters: Δ = 7, 5 ms, ν = μ = 1, 5, N = 100, #trials = 10. The white star denotes the parameter values used in section ‘Heterogeneous networks are more efficient than homogeneous networks’ of the main text. Fig B: Comparison of efficiency measures for the noise simulations Efficiency normalized by amplitude Ea (first column), amplitude squared Ea2 (second column) and cost (third column) of a homogeneous network (top row), a network with ‘type 1’ and ‘type 2’ neurons (middle row) and a heterogeneous network (bottom row). Next to the stimulus, each neuron in the network was presented with a noise input, with a varying relative amplitude (horizontal axis) and number of copies of the noise signal (vertical axis; 1 copy means all neurons receive the same noise, 100 copies means all neurons receive independent noise). Network: Δ = 7, 5 ms, ν = μ = 1, 5, N = 100. Stimulus: amplitude = 10, τ = 15 ms. Noise: τ = 15 ms. (PDF) [file pcbi.1008673.s001.pdf]

Efficient and robust coding in heterogeneous recurrent networks – Supplementary Text and Figures

Fleur Zeldenrust[1*], Boris Gutkin[2,3], Sophie Denéve[2]

1 Department of Neurophysiology, Donders Institute for Brain, Cognition and Behaviour, Radboud University, Nijmegen, the Netherlands
2 Group for Neural Theory, INSERM U960, Département d'Études Cognitives, École Normal Supérieure PSL University, Paris, France
3 Center for Cognition and Decision Making, National Research University Higher School of Economics, Moscow, Russia

* f.zeldenrust@neurophysiology.nl

A A word on notation

Firstly, we use the terms 'filter' and 'kernel' interchangably in this paper. Secondly, following the notation of amongst others Dayan and Abbott [1], we describe a spike-triggered average (STA) or an input filter as a filter where positive time t denotes the time before the spike:

$$\mathrm{STA}(t) = s(-t) * \rho(t) = \frac{1}{n}\sum_{i=1}^{n} s(t^i - t) \tag{1}$$

where the symbol $*$ stands for a convolution, $s(t)$ denotes the input signal and t^i are the spike times of the spike train $\rho(t)$:

$$\rho(t) = \sum_{i=1}^{n} \delta(t - t^i)$$

To make an estimate of the input signal given the output spike train, we use a representing filter $g(t)$:

$$\hat{s}(t) = g(t) * \rho(t) = \sum_{i=1}^{n} g(t - t^i) \tag{2}$$

It has been shown that under certain conditions (a linear-nonlinear model, where circular symmetrical input is filtered by an input filter and put through an exponential threshold [2]), the STA can be used as an input filter, which is equal to the flipped version of the representing filter:

$$\mathrm{STA}(t) = g^{\mathrm{in}}(t) = g(-t)$$

In the context of classical linear-nonlinear poisson neuron (LNP) models [3–5] and GLM models [6–9], the representing filter $g(t)$ will typically be (mainly) acausal, and the STA and input filter causal, since each spike represents the current leading up to that spike. Any causal part of $g(t)$ represents the future prediction that each spike makes, i.e. this represents assumptions of the system about correlations in the input (given that the system has only one form of output, i.e. a spike; this interpretation can change if bursts

are taken into account). An acausal input filter can only be used if (a part of) the future of the input is known. This works well for reconstructing a spike train from a stimulus, when the whole stimulus is known. However, when spike times have to be estimated online, i.e. when the decision to place a spike at time t is made at time t, so the stimulus is only known up to time t, only causal input filters can be used. Therefore, we will use only causal input filters.

B Technical notes

B.1 An ambiguity in the spike rule

The model can overestimate the signal, if the network is very small (only a few neurons) and the delay Δ is the same size or larger than the nonzero part of the filter. Therefore, in the following, we will only use values of Δ that are smaller than the size of the filter. Alternatively, the threshold parameter ν can be adjusted to match the amplitude of the input. The alternative spike rule (5) does not need an adjustment of the threshold, but a neuron using this spike rule does not respond to slowly increasing input, or can respond too late to other inputs. Finally, assumptions about how the input changes can be made.

B.1.1 Ambiguity

The spike rule as introduced in equation (4) of the main text has an ambiguity: there are multiple times at which a spike can be placed so that the error is reduced. As formulated now, with online assessment of the error, the system chooses the spike times at the first moment at which a spike reduces the error. However, sometimes the error can be reduced even more if the system would wait a bit longer. The first spike-time at which a spike would reduce the error is not an optimal choice and can lead to an overestimation of the signal. In this section we will give solutions on how to improve the performance of the system.

B.1.2 Solution 1: choose Δ smaller than the size of the filter

If the delay Δ is larger than the size of the filter, the problem explained above does not occur. This can be understood as follows: the MSE is calculated up to $T + \Delta$. However, if Δ exceeds the time of the filter g, there is no estimate between T and $T + \Delta$, so any non-zero signal will add to the MSE. The system tries to reduce this error by firing more spikes, which results in an overestimation of the signal.

B.1.3 Solution 2: gain modulation

A simple solution to the problem posed before, is increasing the threshold of the system with the parameter ν. If we take ν to be slightly smaller than the average surface of the input filter

$$\nu = \frac{1}{2}\int_0^{T+\Delta} g_m(t-T)^2 dt - \epsilon, \tag{3}$$

the neuron will now spike at the right time. However, this also makes the neuron spike more sparsely and hence make a worse estimation of signals with a small amplitude. The threshold will have to be put even higher for larger-amplitude signals. Therefore, the threshold parameter ν should depend on the shape and amplitude of the input signal (ideally on the amplitude of the input signal convolved with the input filter). This assumes a mechanism of gain modulation that we would have to add to the model.

B.1.4 Solution 3: adjusting the spike rule

A third solution is adjusting the spike rule, so that the system waits with firing a spike to see if the error is reduced more one time step later:

$$\Delta E = E^{\text{no spike at } T}(T+\Delta) - E^{\text{spike at } T}(T+\Delta) > 0 \wedge \frac{d\Delta E}{dt} <= 0 \tag{4}$$

which reduces to

$$V_m > \Theta_m + \nu \wedge \frac{dV_m}{dt} <= 0 \tag{5}$$

This spike rule can induce a bursting response to a step input. The system is now capable of responding optimally to a variety of inputs without the need to adjust a parameter line ν. However, wIth this spike rule, the model is not GLM-like anymore. Moreover, the neuron cannot respond to slowly increasing inputs such as ramps.

B.1.5 Solution 4: make an assumption about the autocorrelation of the input

The overestimation-problem arises, because at time $T+\Delta$, we only know the spikes up to T:

$$\int_{t=0}^{T+\Delta} dt \left(g_k(t-T) \sum_{n=1}^{N} \sum_{t_n^i \neq T} g_n(t-t_n^i) \right) =$$
$$\int_{t=0}^{T+\Delta} dt \left(\sum_{n=1}^{N} g_k(t-T) \sum_{t_n^i < t_k^l} g_n(t-t_n^i) \right) + \int_{t=0}^{t_k^l+\Delta} dt \left(\sum_{n=1}^{N} g_k(t-T) \sum_{t_n^i > T} g_n(t-t_n^i) \right). \tag{6}$$

We can make an estimation for the second term on the right of equation (6). For instance, we can assume that the estimate changes slowly relative to the length of the filter, i.e. that the spike train of the network in $T < t < T+\Delta$ is the spike train of $T-\Delta < t < T$ mirrored. However, $g_k(t-T)g_n(t-t_n^i)$ is typically not symmetric in $t_n^i = T$. If we average over the surface of $g_k(t-T)g_n(t-t_n^i)$ before $t_n^i = T$ to compensate for this, this results in an output filter defined by

$$g_k^{\mathrm{o}} = (1+f_{kk})g_k^{\mathrm{i}} * g_k \tag{7}$$

and the lateral filters received by neuron k from neuron n defined by

$$g_{kn}^{\mathrm{l}} = (1+f_{kn})g_k^{\mathrm{i}} * g_n, \tag{8}$$

where

$$f_{kn} = \frac{\int_{\tau=0}^{T} \int_{t=0}^{T+\Delta} |g_k(t-T)g_n(t-\tau)| dt d\tau}{\int_{\tau=T}^{T+\Delta} \int_{t=0}^{T+\Delta} |g_k(t-T)g_n(t-\tau)| dt d\tau} \tag{9}$$

B.2 Conclusion

The model can overestimate the signal, if the amount of neurons is very small and the delay Δ is the same size or larger than the nonzero part of the filter. Therefore, in this paper, we only used values of Δ that are smaller than the size of the filter. Alternatively, the threshold parameter ν can be adjusted to match the amplitude of the input. The alternative spike rule (5) does not need an adjustment of the threshold, but a neuron using this spike rule does not respond to slowly increasing input, or can respond too late to other inputs. Finally, assumptions about how the input changes can be made.

C Efficiency measures

There is not a single standard measure to calculate eficiency. Therefore, we include here two figures similar to Fig 4 and Fig 5 of the main text, but using different efficiency measures. In particular, we first correct for signal amplitude, i.e. we multiply by the stimulus amplitude Amp, reasoning that a network that spikes less to represent a higher stimulus amplitude is more efficient:

$$E_a = Amp \cdot E = \frac{Amp}{\overline{\text{MSE}} \cdot A}. \tag{10}$$

Similarly, one could reason that the efficiency should scale with the power of the stimulus, i.e. the square of the amplitude

$$E_{a^2} = Amp^2 \cdot E = \frac{Amp^2}{\overline{\text{MSE}} \cdot A}. \tag{11}$$

Finally, one could also express the linear cost of the network activity, by giving each spike and each unit of error a certain cost:

$$C = a \cdot A + b \cdot \overline{\text{MSE}} \tag{12}$$

Parameters a and b need to be chosen in such a way that both network activity and error contribute similarly to the cost C. On the basis of the results in Fig **??** we choose $a = 0.001$ and $b = 1$.

References

1. Dayan P, Abbott LF. Theoretical Neuroscience: Computational and Mathematical Modeling of Neural Systems. MIT Press; 2001.

2. Pillow JW. Likelihood-Based Approaches to Modeling the Neural Code. In: Doya K, Ishii I, Pouget A, Rao RPN, editors. Bayesian Brain: Probabilistic Approaches to Neural Coding. vol. 70. Cambridge, Massachusets: MIT Press; 2007. p. 53–70.

3. Paninski L. Convergence properties of three spike-triggered analysis techniques. Network: Computation in Neural Systems. 2003;14(3):437–64.

4. Simoncelli EP, Paninski L, Pillow JW, Schwartz O. Characterization of Neural Responses with Stochastic Stimuli. In: Gazzaniga M, editor. The Cognitive Neurosciences. MIT Press; 2004. p. 1385.

5. Chichilnisky EJ. A simple white noise analysis of neuronal light. Network: Computation in Neural Systems. 2001;12:199–213.

6. Pillow JW, Shlens J, Paninski L, Sher A, Litke AM, Chichilnisky EJ, et al. Spatio-temporal correlations and visual signalling in a complete neuronal population. Nature. 2008;454(7207):995–9. doi:10.1038/nature07140.

7. Pillow JW, Paninski L, Uzzell VJ, Simoncelli EP, Chichilnisky EJ. Prediction and decoding of retinal ganglion cell responses with a probabilistic spiking model. The Journal of Neuroscience. 2005;25(47):11003–13. doi:10.1523/JNEUROSCI.3305-05.2005.

8. Paninski L. Maximum likelihood estimation of cascade point-process neural encoding models. Network: Computation in Neural Systems. 2004;15(4):243–262. doi:10.1088/0954-898X/15/4/002.

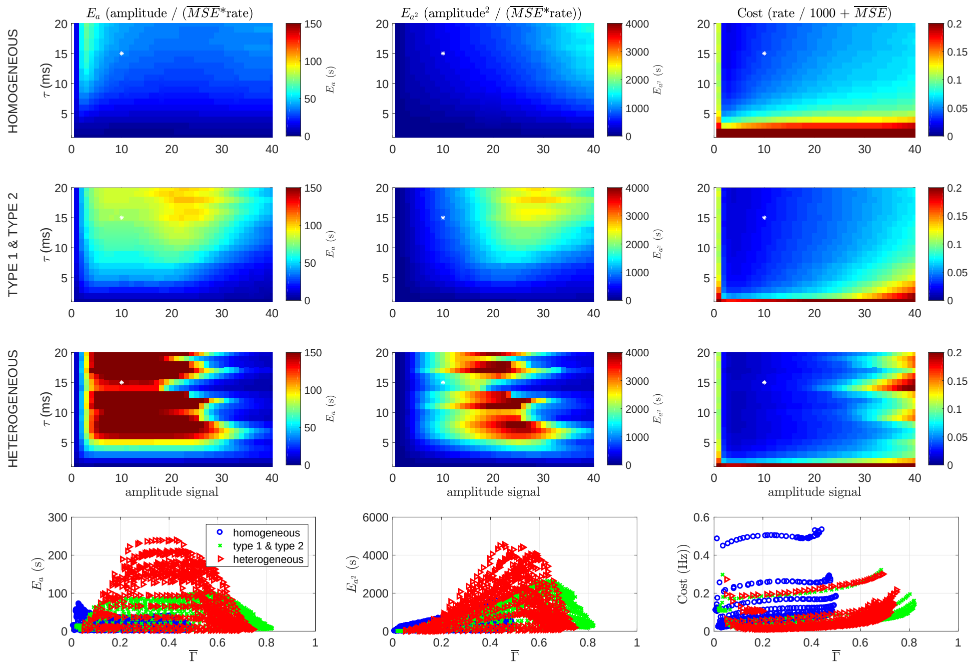

Fig A. Comparison of efficiency measures Results of two simulations using the same stimulus, but different initial network states, in a homogeneous 'type 1' network (first row), a network with 'type 1' and 'type 2' neurons (second row) and a heterogeneous network (third row). Three alternative efficiency measures are compared: 1) the network efficiency multiplied by the stimulus amplitude (E_a, see eq. (10, first column), 2) the network efficiency multiplied by the stimulus power (E_{a^2}, see eq. (11), second column) and 3) the network cost (C, see eq. (12), third column). The bottom row shows how the network efficiency or cost depends on the spike reliability $\overline{\Gamma}$. Parameters: $\Delta = 7,5$ ms, $\nu = \mu = 1,5$, $N = 100$, #trials = 10. The white star denotes the parameter values used in section 'Heterogeneous networks are more efficient than homogeneous networks' of the main text.

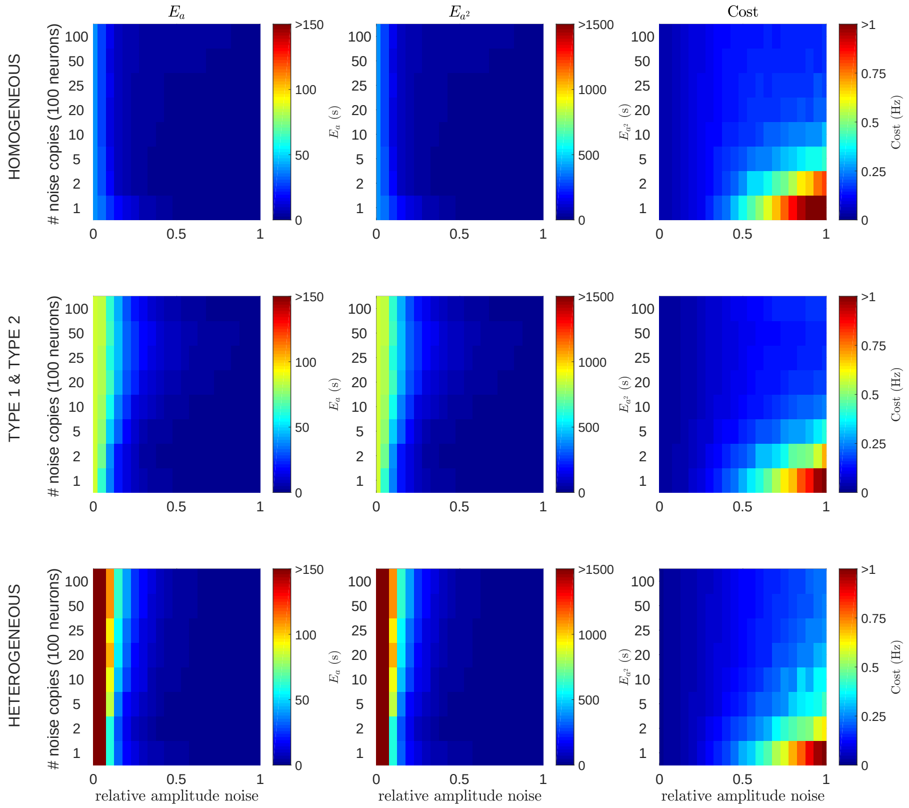

Fig B. Comparison of efficiency measures for the noise simulations Efficiency normalized by amplitude E_a (first column), amplitude squared E_{a^2} (second column) and cost (third column) of a homogeneous network (top row), a network with 'type 1' and 'type 2' neurons (middle row) and a heterogeneous network (bottom row). Next to the stimulus, each neuron in the network was presented with a noise input, with a varying relative amplitude (horizontal axis) and number of copies of the noise signal (vertical axis; 1 copy means all neurons receive the same noise, 100 copies means all neurons receive independent noise). Network: $\Delta = 7,5$ ms, $\nu = \mu = 1,5$, $N = 100$. Stimulus: amplitude $= 10$, $\tau = 15$ ms. Noise: $\tau = 15$ ms.

9. Truccolo W, Eden UT, Fellows MR, Donoghue JP, Brown EN. A point process framework for relating neural spiking activity to spiking history, neural ensemble, and extrinsic covariate effects. Journal of Neurophysiology. 2005;93(2):1074–89. doi:10.1152/jn.00697.2004.
