# Supplementary material for: Efficient and robust coding in heterogeneous recurrent networks
Source: PLoS Comput Biol. 2021 Apr 30;17(4):e1008673. doi: 10.1371/journal.pcbi.1008673 (PMC8115785; doi:10.1371/journal.pcbi.1008673)
Supplement: Attachment — Submitted filename: response reviewers formatted.pdf [file pcbi.1008673.s002.pdf]

DONDERS INSTITUTE

Department of Neurophysiology

Dr Fleur Zeldenrust

Heyendaalseweg 135
Internal mailbox: 33
6525 HJ Nijmegen
The Netherlands
Tel.: +31 (0)24 36 52702

f.zeldenrust@neurophysiology.nl
fleurzeldenrust.nl

Amsterdam, March 18th 2021

Dear editors,

We would like to kindly thank the reviewers for their constructive comments and suggestions. In particular, we value both reviewers comments about the way to measure efficiency and we have added two supplementary figures in which we compare different efficiency measures. We simulated figure 4 with more trials (10), to make the results less dependent on the realization of the choice of random network parameters. We clarified the description of how the thresholds were implemented, and we adapted both introduction and discussion to better clarify what was particular to the framework used here and what was more general, and to include the suggested articles.

Please find a point-by-point summary of how we addressed the reviewer comments below.

Best regards,

Fleur Zeldenrust
Boris Gutkin
Sophie Denève

Reviewer #1:

Major points:

- The argument rests on a model of neurons that produce spikes that perfectly minimise error when doing linear decoding. I think this is an interesting approach, but it might be nice to acknowledge limitations of that approach and discuss to what extent it can be implemented more practically.
  - We have addressed this in the Discussion
- How robust are the results to the choice of efficiency measure? I can see the logic in dividing by number of spikes to get a measure of some sort of accuracy per spike, but 1/MSE as a measure of accuracy doesn't seem totally obvious to me. For example, you might equally argue that there is a cost to each inaccuracy, and a cost for each spike, and you want to minimise the sum of those costs, suggesting that you want to minimise a*num_spikes+b*MSE for some a, b. It would be nice to see that the conclusions about efficiency are robust to the measure chosen here, in the absence of any obvious standard measure.
  - We have added two supplemental figures with several other efficiency measures. Our conclusions were not largely affected by the choice of efficiency measure
- Conclusions are a bit too strongly worded, e.g. "high trial-to-trial variability is a hallmark for an efficiently coding network". In the specific case studied here, yes, but I'm not sure you can generalise this.
  - Adapted in discussion.
- On that specific point (on p8, end of second paragraph of right column), it looks like from Fig 4, bottom right, that actually there isn't quite an inverse relation of E with Gamma, but rather that it peaks around Gamma=0.2?
  - Indeed, we agree with the reviewer, and we have discussed this. It was actually hidden in a footnote (at very low amplitudes, the error is high due to the filters being larger than the stimulus, and Gamma is high). It depends a bit on the efficiency measure used (see supplemental figures), but there appears indeed to be an optimal Gamma.
- Fig 4, heterogeneous MSE panel, what is going on at the right hand side of this figure? Why do some values of tau seem to have (randomly) much smaller or larger errors? Is this noise? Is it feasible to re-run the simulations more times to get a smoother figure?
  - We have done as suggested, and averaged over 10 trials

Minor:

- On p8, left column, there's a combinatorial argument that you would expect the lowest reliability for smallest amplitude signals because the network can choose which neuron to use. But in the simple case of all amplitudes being the same, you would have the maximum number of choices for k where n choose k was largest, i.e. k=n/2. I suspect that I'm making a mistake here though.
  - I am sorry, does the reviewer mean that with this reasoning, the maximal trial-to-trial variability is expected at the stimulus amplitude = # neurons/2 (given that the filter amplitude is 1, since then there would be the optimal number of choices of which neuron can spike)?. I suppose that that is true, and following this reasoning, that would be around an amplitude of about 20 (for 50 ‘positive’ neurons with filters slightly higher than 1). However, this is not taking into account the temporal aspects of the filters, which complicate things: there is ‘leftover’ stimulus estimate from previous spikes, and there are positive and negative parts of a filter. So I agree that this argument is more to gain an intuition than that it completely works out, we have made a comment about that in the text.
- p8, bottom right, some sentences/parts of sentences repeated here.
  - Corrected
- Section numbers are missing throughout.
  - Resolved by formatting everything in PLOS template

Reviewer #2:

Criticism

1. The authors propose a solution to the known "ping-pong" effect of the efficient coding paradigm by adjusting the threshold. Increasing the threshold is a known solution and was proposed in the early works on efficient coding (ref [58] in the paper). In the current paper, the authors define a time-dependent threshold that acts as a filter. This solution is an interesting new view and seems plausible by considering an adaptation of neurons' firing rates. However, I find the explanation of the model lacking. In particular, it is not clear to me how the temporal kernel of the threshold is implemented. Is the kernel convoluted over the inputs or the membrane potential? The authors need to elaborate on how they implement the temporal-kernel threshold. Does it offer benefit when compared to the solution proposed by ref [58]? It is hard to judge the contribution and the biological plausibility without a clear description of the model.

   - The kernel is added to the threshold of neuron m every time neuron m fires a spike, as given in equation (7). The index m had fallen away from this equation, I hope it is clearer now. Adding a neuron-specific temporal increase to the threshold, instead of just an overall one, has as an advantage, that the activity gets 'spread' over multiple neurons, we have added this in the text.

2) The authors devote a large section to the experimental predictions of their theory. The current state of efficient coding theory lacks testable predictions, which will help advance and disseminate the ideas. Here, the authors describe in detail the results of numerical simulations that use biological values for the parameters. Considering it is a simplified model, I could not understand what the actual biological prediction is. The authors indicate that we should not expect to see the expected negative noise correlations because of correlated noise. While important, it is a postdiction of what we do not see in experiments, rather than a testable prediction. I find the experimental predictions the weakest part of this work.
   - We included figure 5 an 6, as these are measurements (signal and noise correlations) that are often done in experiments. Our main argument is exactly that it is difficult to derive definitive conclusions from such measurements, especially on the timescales on which they are measured experimentally. We agree that this is not a conclusive prediction (because such measurements could be in agreement with multiple frameworks or networks). We have introduced the topic differently to make this clearer.

Minor issues

1. The authors note that in deep learning, causal filters are needed because of the layered structure and information flow direction. In my view, causal filters are also needed for a recurrent network. Since the authors are proposing a mechanistic model of neural coding –and not a statistical description– causality seems important for recurrent networks.
   - Indeed our network mechanism is purely causal, i.e. each spike influences the future. However, in post-hoc data analysis, acausal filters are often used as descriptors of neurons (i.e. a spike-triggered average). The text has been adapted to also include recurrent networks.

2. I find some of the definitions slightly confusing:
   1) First, the authors define reliability as the similarity between spike trains. Naturally, one expects reliability to depend on the readout. In that case, a network that can produce the same readout with low error for very different spike trains is more reliable. Counterintuitively, the 'reliability' of the network is very prone to noise. I feel that 'consistency' is a more appropriate term. If the authors choose to keep the term 'reliability,' they may want to emphasize that difference.
      - We understand the confusion, we have called it 'spike reliability'.
   2) The authors do not normalize the efficiency by the signal amplitude, so high-amplitude signals with high activity give low efficiency (which they notice in the results). It is counterintuitive to the meaning of efficiency, and I think it should be pointed out.

- We have added different forms of efficiency in the supplemental materials: normalized by amplitude, power (amplitude squared) and linear cost (as suggested by reviewer 1). Correcting for amplitude (i.e. multiplying by it, as low firing frequencies for high amplitudes would be more efficient than low firing frequencies for low amplitudes), actually made the effects we observed stronger.

3. I could not understand the bottom-left panel of figure 4. Did the authors change the signal strength in different simulations to get different points? Is it just another way of presenting the top panels? I need a more explicit explanation of what is the take-home message from it.

- It is indeed just another way of presenting the top panels. We have included it in the caption.

Typos and errors

1. All the section references are missing.

- Adapted and the paper is now in the PLoS Com Bio template.

2. The authors use the acronyms EPSP and IPSPS but define them only a few sentences after first use.

- Corrected

3. On page 9, the paragraph below the figure, third row. The reference to figure 5 points to the top row, where it should be the bottom row.

- Corrected
